# The molecular mechanism of GADD153 in apoptosis of keloid fibroblasts exposed to botulinum toxin type A

**DOI:** 10.1111/jcmm.16881

**Published:** 2021-09-02

**Authors:** Ming‐Shiuan Nien, Wen‐Pin Cheng, Jun Feng, Yong‐Yan Cui

**Affiliations:** ^1^ Department of plastic surgery Peking University Shenzhen Hospital Shenzhen China; ^2^ Translational Medicine Center Shin Kong Wu Ho Su Memorial Hospital Taipei Taiwan

**Keywords:** apoptosis, Botulinnum Toxin type A, GADD153, keloid fibroblasts

## Abstract

Apoptosis plays a key role in keloids. Growth arrest and DNA damage‐inducible gene 153 (GADD153) is regulated by apoptosis. Botulinum toxin type A (BTXA) can induce apoptosis in keloid fibroblasts. This research aimed to explore the hypothesis that GADD153 mediates apoptosis in keloid fibroblasts exposed to BTXA. BTXA significantly induced GADD153 protein and mRNA expression in keloid fibroblasts. Treatment with c‐Jun N‐terminal kinase (JNK) inhibitor SP600125, JNK small interfering RNA (siRNA) and tumour necrosis factor‐alpha (TNF‐α) antibodies reversed the BTXA‐induced GADD153 expression. BTXA enhanced the transcriptional activity of GADD153, whereas the GADD153 mutant plasmid, JNK siRNA and anti‐TNF‐α antibody treatment abolished the BTXA‐induced transcriptional activity of GADD153. The addition of TNF‐α to keloid fibroblasts markedly increased GADD153 protein expression. The addition of GADD153 siRNA, SP600125 and anti‐TNF‐α antibodies reversed cell death and caspase 3 and 9 activity induced by BTXA.

## 1 BACKGROUND

Wound healing following accidents and medical procedures is an important physiological process that maintains the integrity of the skin.^1^ Proliferative scars caused by abnormal wound healing include hypertrophic scars and keloids.^2^ Both keloids and hypertrophic scars are caused by abnormal chronic inflammation around wounds, and keloid scars often extend beyond the boundaries of the original wound.^3^ A keloid is a type of irregular tissue that is red and hard to the touch, and it often occurs in young people.^4^ A keloid results from fibroblast proliferation and damage to collagen deposition caused by skin injuries, such as those from burns, surgery and other trauma.^5^ Fei et al.^6^ discovered that keloids are benign fibrous tumours caused by abnormally healed skin wounds and that their extracellular matrix accumulates excessively. No universally effective treatment is available for keloids. Keloids are unique to humans and do not exist in animals, and the lack of animal models to simulate the pathogenic process of keloids makes it difficult to study and treat keloids.^7^

Thermal damage induces a loss of calcium in the endoplasmic reticulum (ER), which leads to ER stress.^8^ To reverse the ER stress and maintain the stability of the cell, the ER stress initiates a self‐protection signalling pathway called the unfolded protein response (UPR).^9^ If the ER stress is excessive or the stress response time is too long, the UPR cannot overcome the damage and the cell initiates apoptosis.^10^ ER‐initiated apoptosis is implicated in the pathophysiology of neurodegenerative Parkison's disease,^11^ cardiac hypertrophy,^12^ diabetic cardiomyopathy^13^ and cancer.^14^ C/EBP homologous protein, also known as growth arrest and DNA damage‐inducing protein 153 (GADD153), is the main component of the ER stress‐mediated apoptosis pathway.^15^ As the primary element of the UPR, GADD153 plays two roles in regulating cell survival or cell death.^16^ Under normal circumstances, the expression level of GADD153 in cells is very low, but if this protein is stimulated by genotoxic agents, calcium ionophores, lipopolysaccharides or nutrient deprivation, its expression level increases significantly.^17^ c‐Jun N‐terminal kinase (JNK) and activator protein 1 (AP‐1) play decisive roles in the regulation of GADD153 gene transcription.^18^, ^19^

Apoptosis plays an essential role in keloids.^20^, ^21^ Clinical studies have indicated that botulinum toxin A (BTXA) can induce the apoptosis of keloid fibroblasts and can mitigate hypertrophic scar formation.^22^ Compared with normal adult fibroblasts, keloid fibroblasts have a higher rate of apoptosis in response to hypoxia and interferon‐γ.^23^ It remains unknown whether BTXA can stimulate the expression of GADD153 to induce apoptosis of keloid fibroblasts. In this study, we aimed to investigate 1) whether BTXA can induce GADD153 expression in keloid fibroblasts and 2) what the related molecular regulation mechanisms are. Understanding the effect of BTXA on the expression of GADD153 in keloid fibroblasts may provide new insights for the treatment of keloids.

## 2 MATERIALS AND METHODS

### 2.1 Keloid fibroblast culture

The human keloid fibroblast cell line KEL FIB (ATCC CRL‐1762) was purchased from the American Type Culture Collection (Manassas, VA, USA). Keloid fibroblasts were cultured in Dulbecco's Modified Eagle Medium (DMEM) containing 10% foetal bovine serum (FBS), 100 U/ml penicillin and 100 µg/ml streptomycin at 37℃ under 5% CO2/95% air in a humidified incubator. When confluent, keloid fibroblast monolayers were passaged every 9–10 days after trypsinization and used for experiments from the third to seventh passages. The cells from the third to seventh passages were then cultured in DMEM containing 0.5% FBS, and the keloid fibroblasts were incubated for one additional day to render them quiescent before each experiment was initiated.

### 2.2 Chemicals

BTXA was purchased from Hugel. To determine the roles of JNK, p38 MAP kinase, and p44 MAP kinase in the expression of BTXA‐induced GADD153, keloid fibroblasts were pretreated with SP600125 (20 μM, Calbiochem, San Diego, CA, USA), SB203580 (3 μM, Calbiochem) or PD98059 (50 μM, Calbiochem) for 30 min, respectively, followed by treatment with BTXA.

### 2.3 Western blot analysis

Western blot was performed per the method of a previous study.^24^ In brief, keloid fibroblasts were homogenized in modified RIPA buffer to purify the protein. An equal amount of protein (50 μg) was loaded, and anti‐GADD153 antibodies (1:200 dilution; Santa Cruz Biotechnology) were used. Signals were visualized by chemiluminescent detection. The protein loadings of the samples were further verified to be equal through the staining of monoclonal antibodies against *α*‐tubulin. All Western blots were quantified using densitometry.

### 2.4 Reverse transcription (RT) polymerase chain reaction (PCR)

In brief, total RNA was isolated from keloid fibroblasts using the single‐step acid guanidinium thiocyanate/phenol/chloroform extraction method per a previously described method.^24^

### 2.5 Real‐time quantitative PCR

Real‐time quantitative PCR was performed per a previously described method.^24^ The primers used for real‐time quantitative PCR were as follows: GADD153, 5'‐d(CCCGTGTCGTTCAAAAC)‐3' (forward) and 5'‐d(CGCTGTCCGTGCC GAC)‐3' (reverse), and GAPDH, 5'‐d(CATCACCATCTTCCAGGAGC)‐3' (forward) and 5'‐d(GGATGATGTTCTGGGCTGCC)‐3' (reverse).

### 2.6 Immunohistochemistry

Immunohistochemistry was used to detect the image of GADD153. Antibodies used for immunohistochemistry were anti‐GADD153 antibodies (1:200 dilution; Santa Cruz Biotechnology). Mouse anti‐desmin (1:500; Santa Cruz, Biotechnology Inc., Santa Cruz, CA, USA) was used to mark cytoskeleton. Hochest stain was used to mark the nucleus. We used a confocal microscope to capture the fluorescence signal (Nikon Digital Eclipse; Nikon Instruments, Melville, NY, USA) and used microscope‐related image processing and analysis software for analysis.

### 2.7 Construction of small interfering RNA (siRNA)

Keloid fibroblasts were transfected with GADD153‐annealed siRNA oligonucleotides (Dharmacon Inc., Lafayette, CO, USA) to knock down GADD153 gene expression. The GADD153 targeted base sequences were 5'‐GGUAUGAGGAUCUGCAGGAUU‐3' (sense) and 5'‐P.UCCUGCAGAUCCUCAUACCUU‐3' (antisense), and the JNK1 siRNA were 5'‐CGUGGAUUUAUGGUCUGUGdTdT‐3' (sense) and 5'‐CACAGACCAUAAA UCCACCdTdT‐3' (antisense). For the negative control, dsRNAi was purchased from Dharmacon.

### 2.8 Promoter activity assay

We used T‐Pro NTR II transfection reagent (T‐Pro Biotechnology, Taipei, Taiwan) to transform the GADD153 promoter construct containing the AP‐1 binding site into keloid fibroblasts. The GADD153 promoter‐containing plasmids were designed and transfected into keloid fibroblasts using liposomes as vectors. In addition, the GADD153 promoter construct containing the 5' AP‐1 mutant binding site was also designed and transfected into keloid fibroblasts. Following the treatment of BTXA, keloid fibroblast extracts were prepared using a dual‐luciferase reporter assay system (Promega) and measured for dual‐luciferase activity using a luminometer (Turner Designs).

### 2.9 Detection of TNF‐α concentration by enzyme‐linked immunosorbent assay

In brief, TNF‐α recombinant protein (Calbiochem, San Diego, CA, USA) at different concentrations was added to the cultured medium. The level of IFN‐γ was measured using a quantitative sandwich enzyme immunoassay (Merck Millipore) per a previously described method.^24^

### 2.10 MTT assay

An MTT assay was used to determine keloid fibroblast viability after BTXA treatment. Keloid fibroblasts were adjusted to 3 × 10^4^ cells/ml in DMEM medium. After treatment with BTXA, the medium was aspirated, 0.5 mg/ml MTT (3‐(4,5‐dimethylthiazol‐2‐yl)‐2,5‐diphenyltetrazolium bromide) solution was added, and incubation continued for 4 h. At the end of the incubation period, the MTT solution was removed from the attached cells and the converted dye crystals were dissolved with DMSO. The absorbance of the converted dye was measured at a wavelength of 570 nm.

### 2.11 Cytotoxicity

A cytotoxicity study was performed per a previously described method.^25^ To detect cell injury induced by BTXA, the cell death rate (%) after the addition of BTXA was monitored by trypan blue staining.

### 2.12 Statistical analysis

All results are expressed as the mean ±standard error of the mean. Statistical significance was evaluated in terms of the variance (GraphPad Software, San Diego, CA, USA). Dunnett's test was used to compare multiple groups to a single control group. The Turkey‐Kramer test was used for pairwise comparisons between multiple groups after the ANOVA. A value of *p* < 0.05 was considered statistically significant.

## 3 RESULTS

### 3.1 BTXA increased GADD153 protein and mRNA expression in keloid fibroblasts

**FIGURE 1:**
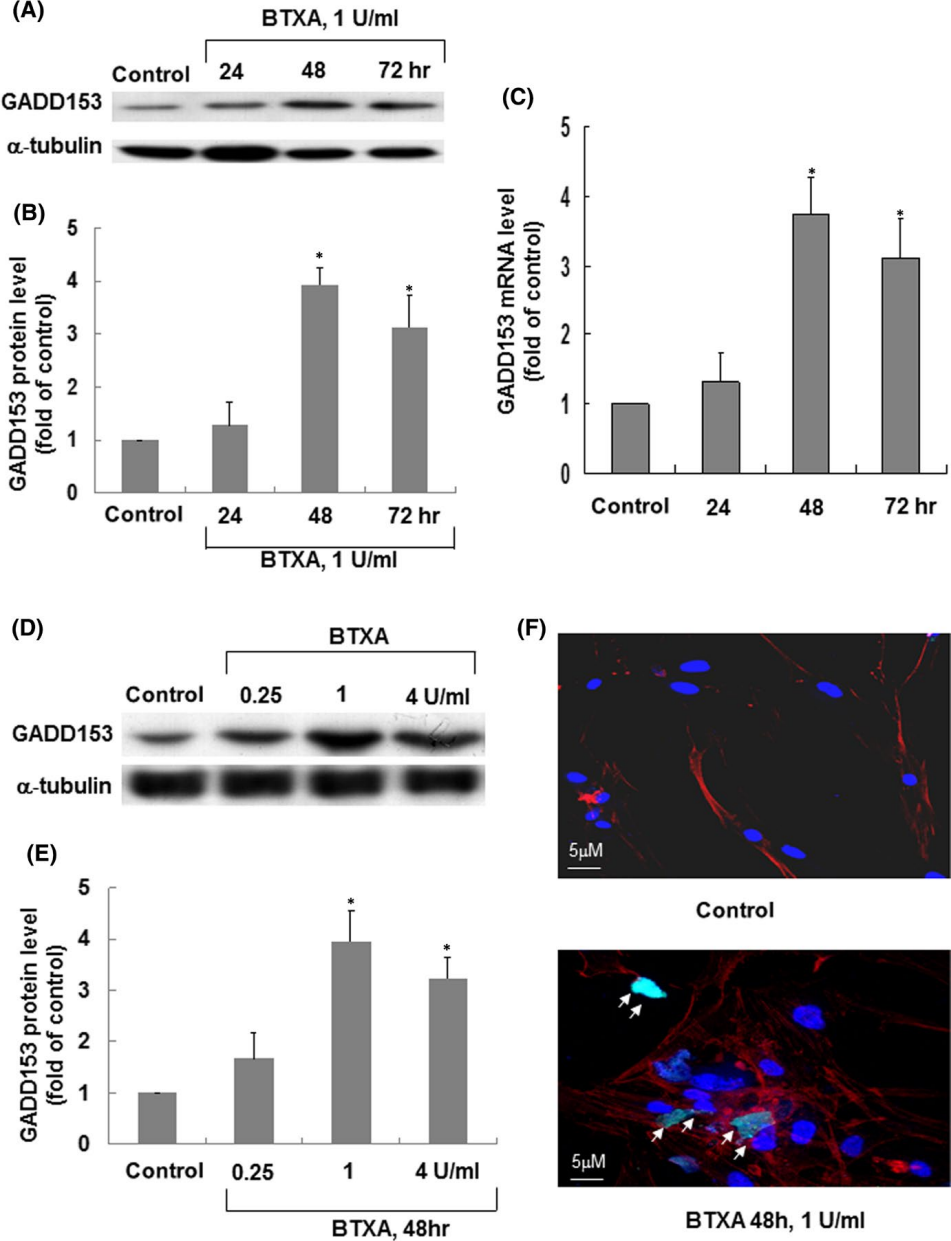
BTXA induces GADD153 protein expression in keloid fibroblasts. (A) Representative Western blots for GADD153 in keloid fibroblasts treated with BTXA (1 U/ml) for various durations. (B) Quantitative analysis of GADD153 protein levels. Values of GADD153 protein treated with BTXA were normalized to match the α‐tubulin measurement and expressed as a ratio of normalized values to protein in the control group. *p < 0.05 versus control. (C) Quantitative analysis of GADD153 mRNA levels. The values of keloid fibroblasts treated with BTXA (1 U/ml) were normalized to match the GAPDH measurement and expressed as a ratio of normalized values to mRNA in the control group (*n* = 3 per group). *p < 0.05 versus control. (D) Representative Western blots for GADD153 in keloid fibroblasts treated with the various doses of BTXA for 48 h. (E) Quantitative analysis of GADD153 protein levels. Values of GADD153 protein levels treated with BTXA were normalized to match the α‐tubulin measurement and expressed as a ratio of normalized values to protein level in the control group. *p < 0.05 versus control. (F) Representative microscopy images of keloid fibroblasts treated with BTXA (1 U/ml) for 48 h. Similar results were observed in another two independent experiments. Arrows indicate GADD153 positive cells

As shown in Figure 1A and 1B, treatment with BTXA (1 U/ml) for 48 h significantly increased the level of GADD153 expression. The real‐time PCR data demonstrated that GADD153 mRNA also increased significantly after treatment with BTXA (1 U/ml) for 48 h (Figure 1C).

We tested the effect of different doses of BTXA (0.25, 1 and 4 U/ml) for 48 h on the expression of GADD153. As shown in Figure 1D, 1E and 1F, BTXA treatment (1 U/ml) significantly enhanced the level of GADD153 expression in keloid fibroblasts. These data indicated that BTXA‐induced GADD153 expression in keloid fibroblasts.

### 3.2 BTXA‐induced GADD153 protein expression in keloid fibroblasts through the JNK pathway

**FIGURE 2:**
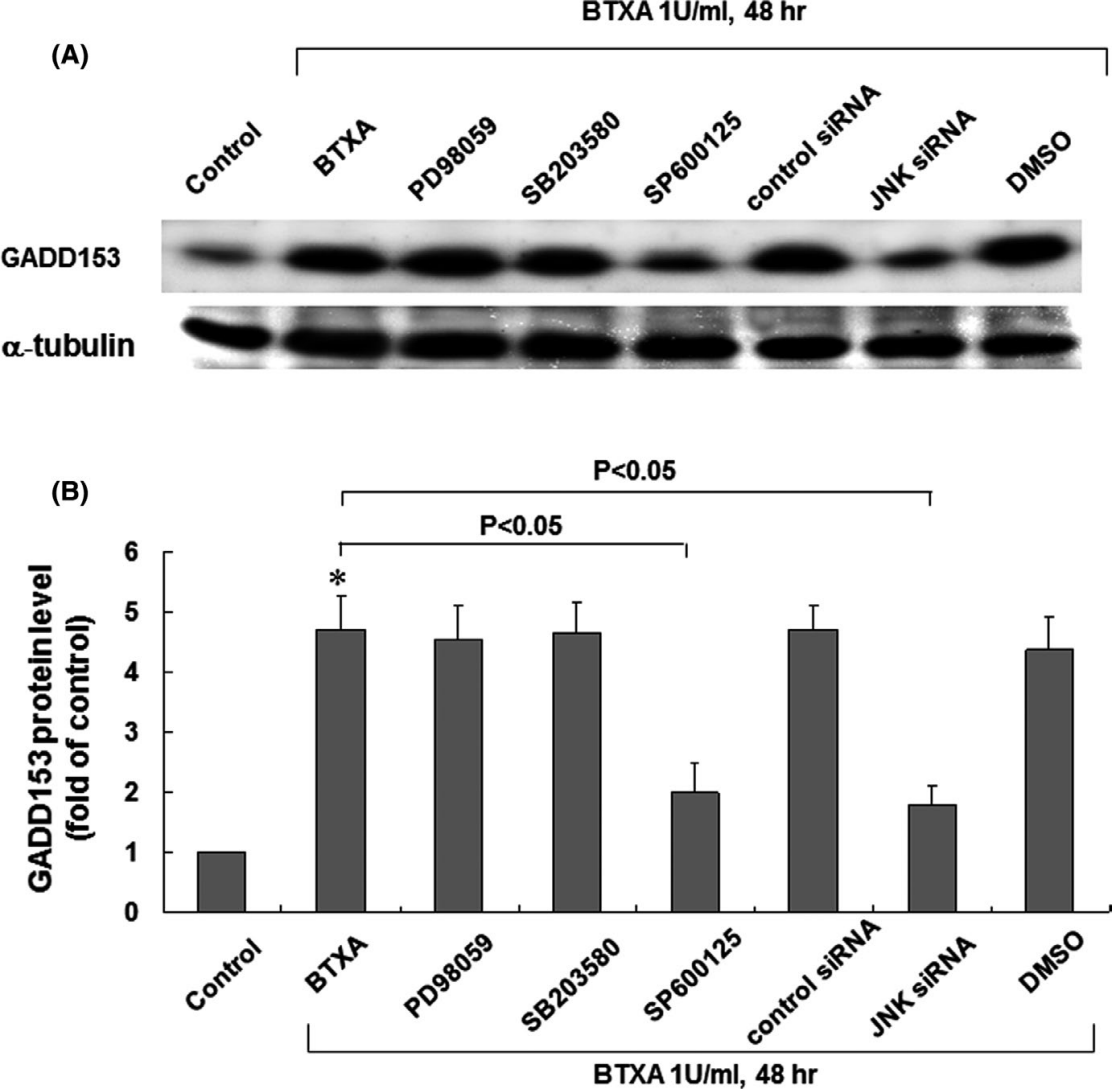
BTXA‐enhanced GADD153 mRNA in keloid fibroblasts through the JNK pathway. (A) Representative Western blots for GADD153 protein levels in keloid fibroblasts treated with BTXA (1 U/ml) in the presence of MAPK inhibitors, JNK small interfering RNA, and vehicle (0.1% dimethyl sulfoxide). (B) Quantitative analysis of GADD153 protein levels. Values of GADD153 protein treated with BTXA (1 U/mL) were normalized to match the *α*‐tubulin measurement and expressed as a ratio of normalized values to protein level in the control group (*n* = 3 per group). *p < 0.05 versus control

GADD153 protein expression induced by BTXA was markedly inhibited when SP600125 (20 μM) and JNK siRNA was added before BTXA treatment (Figure 2). SP600125 is a potent cell‐permeable selective and reversible inhibitor of JNK. GADD153 protein expression induced by BTXA was not affected by the addition of PD98059 (50 μM) or SB203580 (3 μM). PD98059 is a specific and potent inhibitor of ERK kinase, and SB203580 is a highly specific cell‐permeable inhibitor of p38 MAPK. Neither DMSO alone as a vehicle control nor control siRNA affected GADD153 expression induced by hypoxia. These findings suggest that the JNK pathway mediates the induction of GADD153 protein expression by BTXA in keloid fibroblasts.

### 3.3 BTXA enhanced GADD153 promoter activity in keloid fibroblasts

**FIGURE 3:**
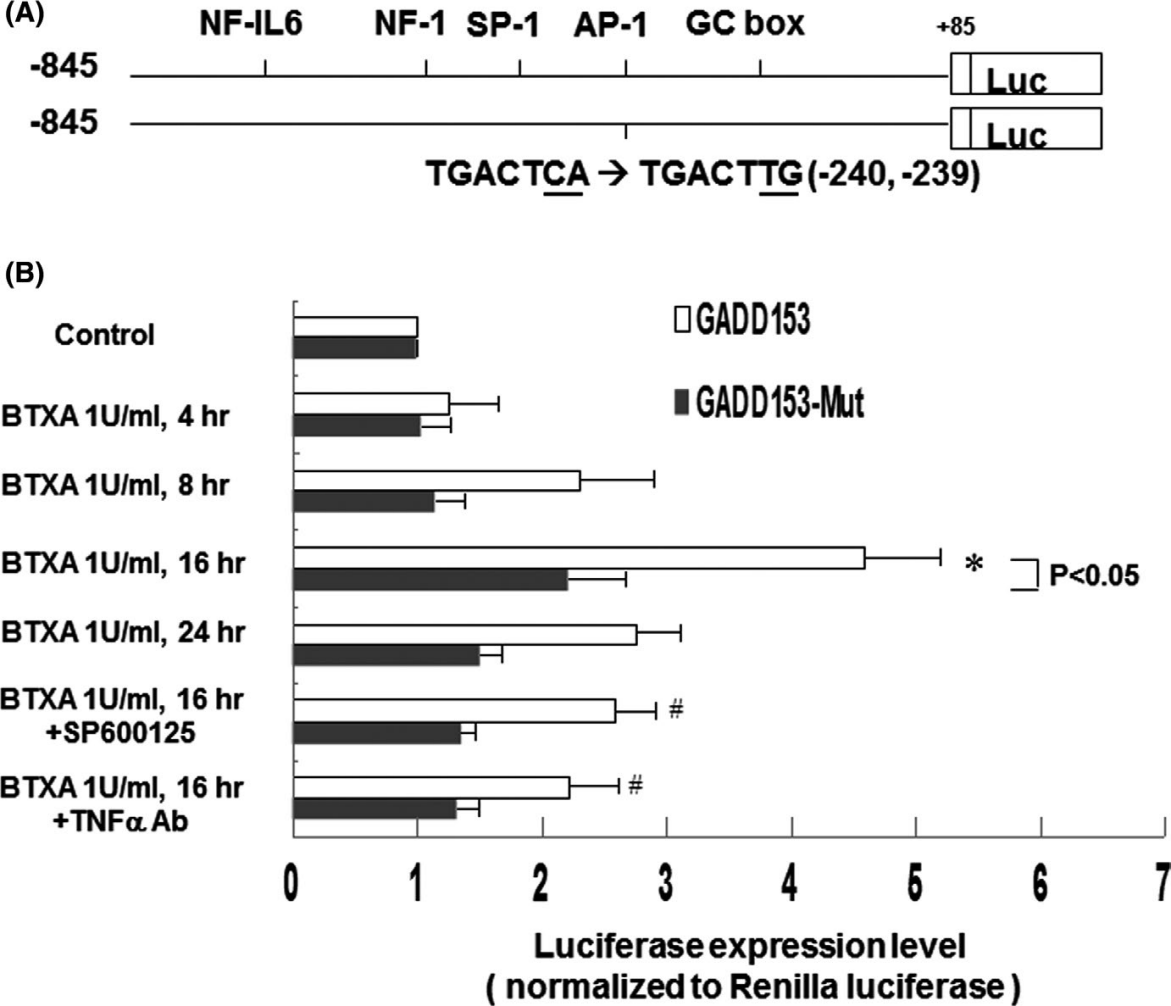
Effect of BTXA on GADD153 promoter activity in keloid fibroblasts. (A) Constructs of GADD153 promoter gene. (B) Quantitative analysis of GADD153 promoter activity of keloid fibroblasts treated with BTXA (1 U/ml) for various durations or with the addition of SP600125 and anti‐TNF‐α antibodies. Keloid fibroblasts were transiently transfected with pGADD153‐Luc using T‐Pro NTR II transfection reagent. The luciferase activity was normalized to renilla activity (*n* = 3 per group). *p < 0.05 versus control. ^#^ *p* < 0.05 versus BTXA 16 h

To investigate whether GADD153 expression induced by BTXA was regulated at the transcriptional level, we used a luciferase reporter assay to investigate the genetic transcription activity of GADD153 in keloid fibroblasts after BTXA treatment. As shown in Figure 3A, the GADD153 promoter construct contained AP‐1 (binding site: TGACTCA), specificity protein 1, nuclear factor 1, nuclear factor interleukin‐6 (NF‐IL6) and GC box binding sites. We also constructed a GADD153 promoter that contained an AP‐1 mutation (binding site: TGACTTG).

As shown in Figure 3B, GADD153 promoter activity increased significantly after BTXA treatment (1 U/ml) for 16 h, but the same effect was not observed for the GADD153 mutant. Treatment with SP600125 and anti‐TNF‐α antibodies before the addition of BTXA reversed the BTXA‐induced promoter activity. These findings suggest that BTXA‐induced GADD153 expression in keloid fibroblasts is mediated by the AP‐1 pathway.

### 3.4 **BTXA‐enhanced GADD153 protein expression in keloid fibroblasts through TNF‐**α

**FIGURE 4:**
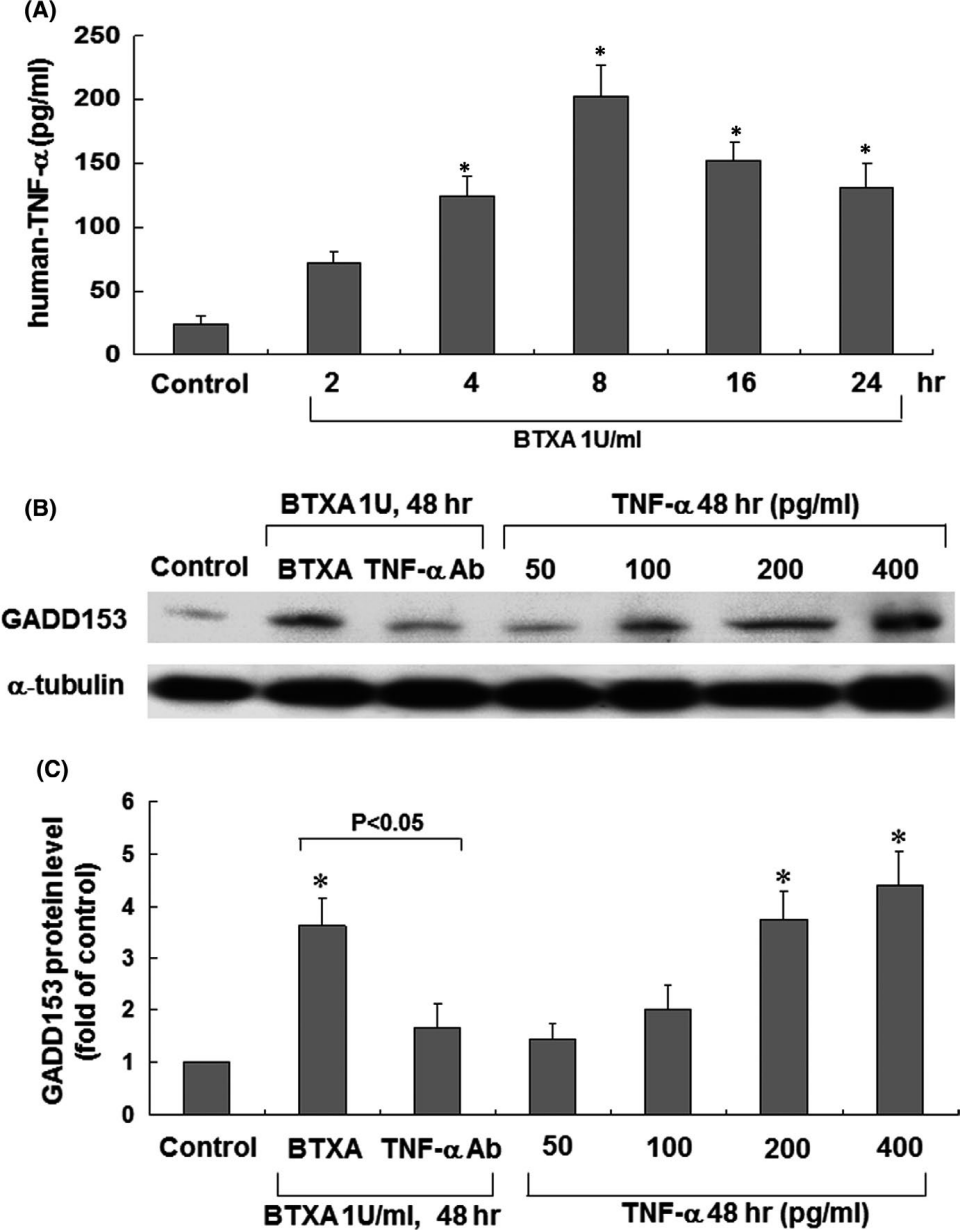
Effects of human TNF‐α on GADD153 in keloid fibroblasts. (A) Release of human TNF‐α from keloid fibroblasts treated with BTXA (1 U/ml) over different durations (*n* = 3 per group). *p < 0.05 versus control. (B) Representative Western blots of GADD153 in keloid fibroblasts after the addition of human TNF‐α or treatment with BTXA (1 U/ml) in the presence of anti‐TNF‐α antibodies. (C) Quantitative analysis of GADD153 protein levels. Values of GADD153 protein were normalized to match the *α*‐tubulin measurement and expressed as a ratio of normalized values to the control cells (*n* = 3 per group). *p < 0.05 versus control

As shown in Figure 4A, TNF‐α secreted from keloid fibroblasts after BTXA treatment markedly increased at 4 h and remained elevated for 24 h. This finding suggests that BTXA increased the secretion of TNF‐α from keloid fibroblasts.

To explore the effect of TNF‐α on GADD153 expression in keloid fibroblasts, we added different concentrations of TNF‐α to the cultured medium and incubated the solutions for 48 h. As shown in Figure 4B and C, the effect of the exogenous administration of TNF‐α on GADD153 protein expression in keloid fibroblasts was dose dependent. Furthermore, the addition of anti‐TNF‐α antibodies 30 min before BTXA treatment significantly reduced BTXA‐induced GADD153 protein expression. These results indicate that TNF‐α‐mediated GADD153 protein expression in keloid fibroblasts treated with BTXA.

### 3.5 **BTXA‐enhanced GADD153 protein expression in keloid fibroblasts through TNF‐**α

**FIGURE 5:**
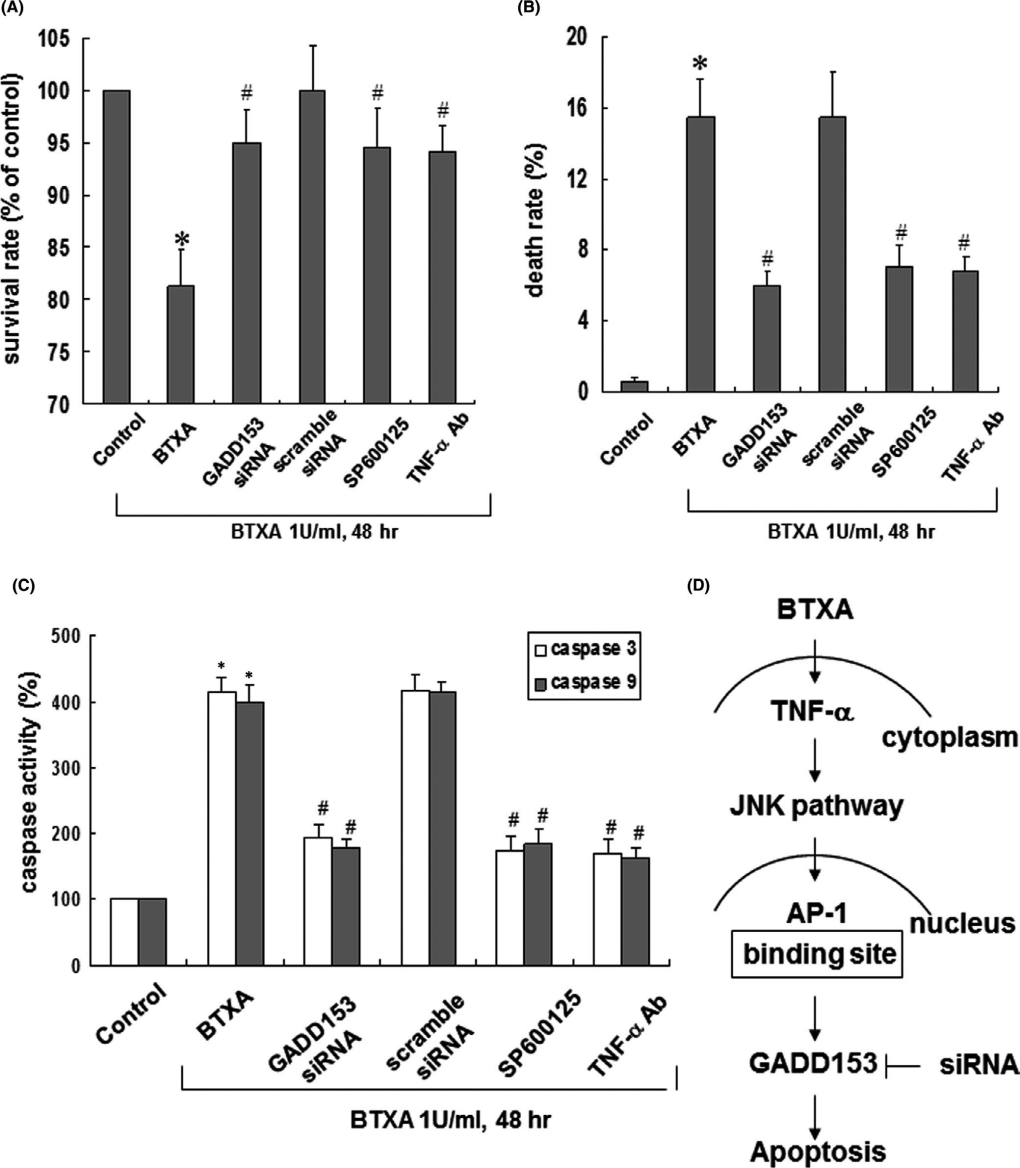
Effect of GADD153 on BTXA‐induced apoptosis in keloid fibroblasts. Quantitative analysis using MTT assay (A) and trypan blue exclusion (B) to determine the viability of keloid fibroblasts treated with BTXA (1 U/ml) in the presence of GADD153 siRNA, SP600125 or anti‐TNF‐α antibodies (*n* = 3). *p < 0.05 versus control. (C) Quantification of caspase 3 and 9 activity in keloid fibroblasts induced by treatment with BTXA (1 U/ml) in the presence of GADD153 siRNA, SP600125 or anti‐TNF‐α antibodies. *p < 0.05 versus control. ^#^ *p* < 0.05 versus BTXA 48 h. (D) Schematic diagram of BTXA‐induced GADD153 regulatory pathway

GADD153 siRNA was used to investigate the role of GADD153 in cell death and apoptosis after BTXA treatment. BTXA reduced the viability of keloid fibroblasts, and this effect was eliminated after treatment with GADD153 siRNA, SP600125 and anti‐TNF‐α antibodies (Figure 5A). As shown in Figure 5B, pretreatment with GADD153 siRNA, SP600125 and TNF‐α antibodies prior to BTXA treatment reversed the BTXA‐induced increase in the cell death rate of keloid fibroblasts. These results suggest that BTXA‐induced cell death in keloid fibroblasts. Additionally, we determined the caspase 3 and 9 activity to assess apoptosis of keloid fibroblasts after BTXA treatment. Our result demonstrated that the addition of GADD153 siRNA, SP600125 and TNF‐α antibodies reversed the caspase 3 and 9 activity induced by BTXA. These findings indicate that GADD153 mediated the BTXA‐induced apoptosis of keloid fibroblasts.

## 4 DISCUSSION

This research presents several key findings. First, BTXA induced the expression of GADD153 protein and mRNA in human keloid fibroblasts. Second, when cells were treated with BTXA, JNK MAP kinase and AP‐1 transcription factors were both related to the GADD153 signalling pathway. Third, BTXA increased the expression of TNF‐α in keloid fibroblasts. Fourth, BTXA‐activated GADD153 protein expression in keloid fibroblasts via the TNF‐α pathway. Fifth, BTXA induced the death and apoptosis of keloid fibroblasts via GADD153. Finally, BTXA increased the expression of GADD153 in both a time‐ and dose‐dependent manner.

We demonstrated that BTXA activates the expression of GADD153 protein via the JNK MAPK pathway in keloid fibroblasts. SP600125, a specific inhibitor of JNK, abolished the GADD153 protein expression induced by BTXA. However, specific inhibitors of ERK and p38 did not affect BTXA‐induced GADD153 protein expression. Yun et al. reported that the host's response to BTXA is dependent on toll‐like receptor 2 (TLR2), which is regulated by the JNK pathway.^26^ In addition, another study demonstrated that 1) BTXA inhibited the formation of scar‐related factors and the extracellular matrix in human scar fibroblasts and 2) the JNK pathway had a regulatory effect in this process.^27^ These results suggest that BTXA is closely related to the regulation of JNK MAPK.

GADD153 promoter activity requires AP‐1, the downstream target of JNK.^28^ Through reporter gene testing, we demonstrated that the BTXA‐induced transcriptional activity of the GADD153 promoter is AP‐1 dependent. Our results indicate that the JNK pathway is the main pathway through which BTXA induces GADD153 expression. Previous studies have demonstrated that genistein exerts antiproliferative and proapoptotic effects on the expression of AP‐1 subunits in keloid fibroblasts.^29^ Wei et al.^30^ suggested that a single complex, which contains AP‐1 and SMAD binding complex components, is responsible for responding to serum transactivation. These aforementioned studies have indicated that the regulation of AP‐1 plays a key role in keloid fibroblasts. However, previous research has demonstrated that the keloid regression induced by a flash lamp pulsed dye laser is mediated by the blockade of AP‐1 transcription.^31^ This discrepancy may be explained by the different experimental models used between studies. However, these conflicting results demonstrate that the regulatory role of AP‐1 in keloid fibroblasts is still controversial.

Our results indicate that BTXA enhances the expression of TNF‐α in keloid fibroblasts. Previous research has also reported an increased gene expression of TNF‐α in keloid tissues.^32^ In addition, our findings indicate that TNF‐α is involved in the expression of GADD153 induced by BTXA in keloid fibroblasts. Fu‐Tao et al. reported that GADD153 and the TNF‐α pathway were involved in apoptosis induced by ofloxacin in juvenile canine chondrocytes.^33^ These results are consistent with our findings. Moreover, our results revealed that cell death and apoptosis induced by BTXA were eliminated by anti‐TNF‐α antibodies. However, Qijie et al.^34^ demonstrated that TNF‐α can promote the proliferation of keloid fibroblasts. Our results indicated that BTXA‐induced GADD153 expression was mediated by TNF‐α. On the other hand, transforming growth factor β1 (TGF‐β1) is the most common cytokine that promotes scar formation and fibrosis. Zhibo et al.^35^ demonstrated that BTXA can significantly inhibit the growth of fibroblasts derived from hypertrophic scars, thereby causing a decrease in TGF‐β1 protein. Previous study also reported that BTXA can regulate the expression of TGF‐β1 and extracellular matrix protein to inhibit the proliferation of fibroblasts.^36^

In this study, we demonstrated that the BTXA‐induced cell death and apoptosis of keloid fibroblasts is mediated by GADD153. This is finding is consistent with that of Zhanying et al.^37^ that BTXA inhibits the proliferation of human skin keloid fibroblasts and promotes apoptosis by regulating ZEB2 targeting miR‐1587/miR‐2392. In addition, Gil et al. suggested that BTXA reduced the proliferation of human scar fibroblasts.^27^ In this study, we demonstrated that the BTXA‐induced apoptosis of keloid fibroblasts is regulated by JNK. Previous research has indicated that the inhibitory effect of BTXA in hypertrophic scar fibroblasts is closely related to the regulation of JNK.^27^ We further demonstrated that BTXA‐induced caspase 3 and 9 activity and that the addition of GADD153 siRNA reversed such BTXA‐induced caspase 3 and 9 activity. Apoptosis can be divided into intrinsic and extrinsic pathways. Caspase 9 is related to the intrinsic pathway, which in turn induces the effect of caspase3, and ultimately leads to apoptosis. ER stress and GADD153 induces apoptosis through endogenous pathways.^38^ Our results indicated BTXA‐induced keloid fibroblasts apoptosis is through caspase 9 and caspase 3. On the other hand, CACNA1G‐AS1 was previously reported to inhibit the expression of miR‐205, promoting the inhibition of caspase 3 activity in human keloid fibroblasts.^21^ Furthermore, Hao et al.^39^ demonstrated that the inhibition of microRNA‐21 increased caspase 3 and 9 activity in keloid fibroblasts. These studies have indicated that caspase 3 and 9 are closely related to the apoptosis of keloid fibroblasts. The present study suggests that GADD153 may be involved in the apoptosis of keloid fibroblasts treated with BTXA.

In summary, this study is the first to present evidence that BTXA induces GADD153 expression in cultured human keloid fibroblasts. BTXA induces GADD153 expression through TNF‐α, JNK and the AP‐1 pathway.

## CONFLICTS OF INTEREST

The authors declare that they have no conflicts of interest.

## AUTHOR CONTRIBUTIONS

**Ming‐Shiuan Nien:** Funding acquisition (equal); Methodology (equal); Resources (equal); Writing‐original draft (equal). **Wen‐Pin Cheng:** Investigation (equal); Methodology (equal); Resources (equal); Writing‐original draft (equal); Writing‐review & editing (equal). **Jun Feng:** Methodology (equal); Resources (equal). **Yong‐Yan Cui:** Funding acquisition (equal); Resources (equal); Supervision (equal).
